# A Multicenter Survey on Pharmacists’ Perspectives on Self-Medication Issues in Romania: A Descriptive Study Towards Sustainable and Safe Pharmaceutical Practices

**DOI:** 10.3390/healthcare12222316

**Published:** 2024-11-20

**Authors:** Victor Gheorman, Flavius Cristian Mărcău, Cătălin Peptan, Veronica Gheorman, Marian Emanuel Cojoaca, Alina Magdalena Musetescu, Mitutoiu Daniela, Felicia Militaru

**Affiliations:** 1 Department of Psychiatry, University of Medicine and Pharmacy of Craiova, 200349 Craiova, Romaniafelicia.militaru@umfcv.ro (F.M.); 2 Department of Psychiatry I, Craiova Clinical Neuropsychiatry Hospital, 200473 Craiova, Romania; 3 Faculty of Educational Sciences, Law and Public Administration, “Constantin Brâncuși” University of Târgu Jiu, 210185 Târgu Jiu, Romania; flavius.marcau@utgjiu.ro (F.C.M.);; 4 Department of Medical Semiology, University of Medicine and Pharmacy of Craiova, 200349 Craiova, Romania; 5 National Health Insurance House (CNAS), Titu Maiorescu University, 040051 Bucharest, Romania; 6 Victor Babeș Hospital for Infectious and Tropical Diseases, Titu Maiorescu University, 040051 Bucharest, Romania; 7 The Faculty of Entrepreneurship, Engineering, and Business Management, National University of Science and Technology Polytechnic Bucharest, 060042 Bucharest, Romania; dana.mitutoiu@upb.ro

**Keywords:** pharmacist, self-medication, health, sustainable development, medical security

## Abstract

Background/Objectives: This study evaluates the perceptions of pharmacists in Romania regarding self-medication, a growing practice with significant implications for public health and sustainable development. The main objective is to analyze the role of pharmacists in managing self-medication and educating the public on rational medication use. Methods: A descriptive and exploratory methodology was employed, using a questionnaire administered to 300 pharmacists from various regions in Romania between March and April 2024. The data analysis included Chi-square tests and linear regression to identify predictive factors. Results: The findings reveal a direct correlation between the frequency of self-medication and the perceived severity of conditions, with self-medication being more common for minor ailments. Experienced pharmacists are less likely to encourage self-medication. Public education on the risks of self-medication significantly reduces the prevalence of this practice. Conclusions: Self-medication, without proper regulation and education, risks becoming unsustainable. Targeted informational campaigns and educational programs tailored to diverse demographic groups are essential. Pharmacists play a critical role in promoting safe and responsible medication use.

## 1. Introduction

Self-medication, the practice of using medications without professional guidance, is prevalent in Romania due to the easy accessibility of over-the-counter (OTC) drugs and widespread health information, often lacking verification. Self-medication is a common practice globally, influenced by various factors such as access to healthcare services, the patient’s place of residence, and perceptions of disease management. While this practice may offer benefits, such as the rapid alleviation of minor symptoms and reducing pressure on the healthcare system, it is also associated with significant risks. These risks include misdiagnoses, inappropriate dosing, adverse drug interactions, and, most notably, the improper use of antibiotics, which contributes to the development of antimicrobial resistance [1,2,3,4,5]. In Romania, self-medication shows significant variations between urban and rural areas [6,7,8,9,10].

In recent years, the influence of social networks and the internet [11] has played a significant role in shaping self-medication behaviors [12]. Although these platforms have the potential to enhance health awareness and patient education, they also spread misinformation, which can lead to incorrect self-treatment. Therefore, it is crucial to implement health education programs, well-targeted public campaigns, and preventive strategies to ensure informed, safe, and rational self-medication practices across different demographic groups in Romania [13]. The adequate education of the population on the benefits and dangers of self-medication should be integrated both into the school curriculum to develop health literacy and through interventions provided by healthcare professionals in settings such as pharmacies or medical consultations. Such education can support the safe and rational use of medications, promoting informed decision making. Implementing well-targeted public health campaigns that provide practical and relevant information on self-medication can contribute to improving health behaviors and reducing irrational or dangerous self-medication practices, thereby contributing to a more efficient and safer healthcare system [12], which serves as a foundation for ensuring state security.

This study explores pharmacists’ perceptions and roles in managing self-medication, focusing on its drivers such as public education and professional experience [14,15,16]. Pharmacists act as critical gatekeepers, helping to ensure the safe use of OTC drugs and guiding public health practices. Understanding their approaches is key to developing informed public health strategies, educational programs, and policies to mitigate the risks associated with self-medication, particularly those related to antimicrobial resistance and improper medication use [16,17,18,19,20,21].

### 1.1. Hypotheses

Thus, our study is based on the following hypotheses:

**Hypothesis** **1** **(H1).** *There is a direct correlation between the frequency of self-medication and the perceived severity of the condition, with a higher number of self-medication cases for conditions considered minor.*

**Hypothesis** **2** **(H2).** *The urban population is more likely to practice self-medication compared to the rural population, possibly due to easier access to medical services and health information.*

**Hypothesis** **3** **(H3).** *Pharmacists with greater professional experience are less likely to recommend/encourage self-medication compared to their less experienced colleagues, due to a better understanding of the associated risks.*

**Hypothesis** **4** **(H4).** *The better education of the population about the risks and safety of medications can significantly reduce the prevalence of unjustified self-medication, with a positive impact on the health of the population and the sustainable development of global society.*

### 1.2. Objectives

Additionally, the objectives of our study are as follows:

Objective 1 (O1): Establishing the relationship between the frequency of self-medication and the perception of the severity of conditions.

Objective 2 (O2): Identifying the differences in perception regarding self-medication between urban and rural pharmacists.

Objective 3 (O3): Evaluating the impact of pharmacists’ professional experience on self-medication recommendations.

Objective 4 (O4): Highlighting the role of education in changing self-medication behaviors and its impact on the health of the population and the sustainable development of global society.

## 2. Literature Review

Self-medication practices, although common globally, have implications for social security, especially when considering access to medical care and personal safety [22,23]. The review explores various dimensions of self-medication (globally, but also in Romania), focusing on different segments of the population [5,24,25,26], motivational factors [27,28,29], and results associated with these practices [30,31,32]. Such behaviors are significantly influenced by easy access to over-the-counter medications and are often exacerbated by aggressive marketing strategies and an insufficient and permissive regulatory framework [33,34].

Self-medication in different age groups of the Romanian population reveals specific patterns and factors that influence this behavior [4,23,35,36].

In rural areas of Romania, the lack of health services and greater reliance on self-medication generate significant health risks, especially in situations where patients avoid or do not have access to specialist medical advice, preferring the advice and recommendations of pharmacists, which are more accessible and temporally efficient [5,37]. This behavior indicates broader issues related to accessibility to medical care, which can impact social security by increasing health risks [38,39].

The widespread practice of self-medication presents risks such as antimicrobial resistance and drug interactions, necessitating interventions to educate the population and reduce the irrational use of medications [40,41]. This is essential in maintaining social health security and ensuring that future generations can manage health autonomously and safely.

Self-medication in Romania is influenced by a variety of factors, such as the population’s poor medical culture, cultural habits, and accessibility to medical care [42,43,44], highlighting a discrepancy between knowledge and action, an aspect crucial for health policy formulation. Although it allows individuals to manage minor health problems, the associated risks require targeted education and health sector reforms to protect social security.

The issue of managing the self-medication of the population, imperatively necessary to ensure the health of the population, seen as an essential condition for the sustainable development of society at the global level, has recently been addressed in the specialized literature, from the perspective of preventing and treating substance abuse, including drugs [45,46,47]; ensuring universal coverage with health services [48,49,50]; or ensuring access to drugs at affordable prices [51,52,53]. Also, specialized studies highlight the role of pharmaceutical personnel, from the sensor perspective, in issuing early alerts, in order to reduce and manage the risks to the health of the population [54,55,56] and contribute substantially to the sustainable development of society, including regarding human security, considering the importance of population health in its architecture [57,58,59].

## 3. Materials and Methods

### 3.1. Research Design

This study adopts a descriptive and exploratory approach to investigate the perceptions and behaviors of pharmacists in Romania regarding self-medication. A questionnaire was used to assess pharmacists’ experiences with self-medication practices, frequency of interactions, and strategies in managing self-medication requests. The goal was to provide a comprehensive understanding of how pharmacists approach self-medication in the Romanian context.

### 3.2. Sample Selection

A stratified probabilistic sampling method was used to select a representative sample of 300 pharmacists from various urban and rural areas, as well as from different types of pharmacies (independent and chain). The inclusion criteria required pharmacists to be actively practicing, with at least six months of experience in a pharmacy setting, to ensure familiarity with the dynamics of daily pharmaceutical practice. The questionnaire was distributed to all pharmacist colleges across Romania. Despite the limited number of participants, the respondents are geographically distributed throughout the entire country, providing a diverse range of perspectives while maintaining relevance to the study’s objectives. Pharmacists without direct client interaction or those on leave were excluded.

### 3.3. Data Collection

Data were collected via an online questionnaire distributed through professional networks. The questionnaire was designed to evaluate pharmacists’ perceptions, behaviors, and experiences related to self-medication. Pilot testing was conducted with a group of 20 pharmacists to ensure clarity, functionality, and validity. This group was selected to represent diverse levels of professional experience, allowing for relevant feedback on question formulation and technical aspects. Additionally, feedback from experts in pharmacy and public health further validated the content and structure of the questionnaire.

### 3.4. Data Analysis

The data were preprocessed and analyzed using SPSS version 26. Descriptive statistics provided an overview of responses. A Kaplan–Meier survival analysis and multiple linear regression were applied to understand the time-to-event data and identify factors influencing self-medication practices. Chi-square tests were used to detect significant differences between groups. The analysis primarily aimed to identify trends and predictive factors without overwhelming the reader with methodological specifics.

### 3.5. Ethical Considerations

Ethical approval was obtained following national and international standards, including the Declaration of Helsinki and GDPR guidelines. Participants were informed of the study’s purpose, and consent was gathered electronically before the completion of the questionnaire, ensuring anonymity and voluntary participation.

## 4. Results

### 4.1. Socio-Demographic Characteristics and Pharmacists’ Context

Table 1 presents the socio-demographic structure of the group of respondents (pharmacists) involved in the study, highlighting the diversity in terms of the professional experience, work environment, age, education level, and type of pharmacy they work in. The majority of the surveyed pharmacists (64.6%) have more than 10 years of professional experience, work in urban areas (85.0%), are employed in pharmacy chains (58.0%), and have university or postgraduate degrees (98.2%). There is a balanced distribution of respondents under the age of 40 (51.0%) and those over 40 (49.0%). The profile of the respondents suggests a solid base of professional experience, life experience, and social interaction, relevant for addressing the phenomenon investigated in this study.

### 4.2. The Frequency and Typology of Self-Medication Based on Conditions and Medications Used

Table 2 highlights the dynamics of self-medication requests and the types of medications used for various medical conditions. The most frequent self-medication requests pertain to minor conditions, where 92.3% of pharmacists reported a daily request for medications, with the majority of patients opting for analgesics (23.9%). In contrast, for more serious conditions, self-medication is significantly reduced (59.3%); however, it is concerning that antibiotics (19.1%) are frequently used without a medical prescription, raising concerns about their overuse.

The data highlight the dynamics of self-medication practices across different conditions. Minor conditions are self-medicated daily, with analgesics being the most common choice (23.9%), showing significantly higher frequencies than expected (*p* < 0.001). In contrast, serious conditions, despite being less frequently self-medicated (59.3%), raise concerns due to the overuse of antibiotics (19.1%, *p* < 0.001). Self-medication for colds and flu is prevalent (65.0%), often involving anti-inflammatory drugs (23.2%, *p* < 0.001). Gastrointestinal conditions and sleep or stress disorders show lower frequencies of self-medication, with antipyretics (11.8%) and anxiolytics (7.2%), respectively, being less requested than expected. COVID-19-related self-medication is occasional, mainly involving dietary supplements (14.1%), with no significant deviations from expected patterns.

### 4.3. Access to Medication Information and Influences on Self-Medication Behavior

Table 3 provides an overview of pharmacists’ perceptions regarding access to medication information, the factors influencing patients’ self-medication behavior, and the quality of information available in pharmacies.

The data illustrate key perceptions regarding access to medication information, influences on self-medication behavior, and the quality of information in pharmacies. Only 26.3% of pharmacists reported that patients consistently have access to medication information, with 43.3% noting occasional access, which may contribute to variability in self-medication decisions. Medication advertising significantly influences self-medication behaviors, as highlighted by 78% of pharmacists, followed by information from social networks (50%), demonstrating the impact of informal information sources. Regarding the quality of information provided in pharmacies, 45% of pharmacists rated it as “high” and 38% as “very high,” though 2.3% acknowledged areas for improvement, citing “low quality” in some cases.

Table 4 presents pharmacists’ perceptions regarding the impact of the COVID-19 pandemic on the phenomenon of self-medication.

The majority of pharmacists reported that the pandemic significantly influenced patients’ self-medication behaviors. This suggests that during the pandemic, patients were more inclined to resort to self-medication, either due to limited access to medical services or fear of visiting healthcare facilities. On the other hand, a significantly smaller percentage of pharmacists believe that the pandemic had no effect on self-medication.

### 4.4. Pharmacists’ Practices in Managing Self-Medication and Improvement Proposals

Table 5 provides a detailed overview of pharmacists’ perceptions regarding the use of over-the-counter (OTC) medications, the level of awareness about the risks associated with self-medication, and the comfort level of pharmacists in handling inappropriate medication requests.

The data in Table 5 shed light on pharmacists’ understanding of non-prescription medication use, patients’ risk awareness, encouragement of medical consultation, and pharmacists’ comfort in managing self-medication. A significant portion of pharmacists (43.3%) believe that only 25–50% of OTC medication requests are justified, reflecting caution in recommending these medications. However, patients’ awareness of self-medication risks is low, with 59.3% of pharmacists reporting very low or low awareness, underscoring the need for better patient education. Encouragingly, 89% of pharmacists consistently recommend medical consultations for serious conditions, highlighting their role in ensuring safe practices. Despite this, 52.6% of pharmacists feel very comfortable managing requests for inappropriate medications, suggesting confidence in addressing unjustified patient demands.

Table 6 provides insights into the existence of procedures for evaluating the necessity of non-prescription medications and how pharmacists manage requests for inappropriate medications in self-medication.

The findings reveal that 48.6% of pharmacists implement procedures for evaluating non-prescription medications only in some cases, highlighting an inconsistent application of protocols. A smaller proportion, 37.3%, consistently apply such procedures, while 14% report no formal protocols, suggesting a need for standardized approaches.

In response to inappropriate medication requests, 56.6% of pharmacists recommend medical consultation, emphasizing their focus on patient safety. Additionally, 37.6% provide education on the risks of self-medication, underscoring their role in raising patient awareness. Only 5% of pharmacists refuse requests outright, indicating that direct rejection is rare, with most preferring education or referral to healthcare professionals. Lastly, 0.6% of pharmacists use other methods to manage these situations, further emphasizing the preference for consultation and education as primary strategies.

Table 7 highlights how pharmacists manage risky self-medication and their proposed improvements.

Table 8 presents a detailed analysis of demographic and professional factors influencing self-medication practices, highlighting the role of various independent variables in self-medication in different contexts. The interpretation of each relationship is based on unstandardized and standardized regression coefficients, as well as *p*-values, which indicate the statistical significance of these relationships.

Table 8 highlights the demographic and professional factors influencing self-medication practices. Self-medication for sleep and stress disorders is reported more frequently in independent pharmacies (*p* = 0.006), suggesting that such environments may facilitate these behaviors. For COVID-19-related self-medication, urban pharmacies show significantly lower frequencies than rural ones (*p* = 0.028), and pharmacists with higher education levels report a reduced prevalence of such behaviors (*p* = 0.044).

Age also plays a role, as younger pharmacists observe higher levels of self-medication for colds and flu (*p* = 0.001) and perceive social networks as having a greater influence on these behaviors, although this is less significant (*p* = 0.080). Professional experience mitigates the impact of advertisements on self-medication decisions (*p* < 0.001) and correlates with fewer requests for online-sourced medication information (*p* = 0.005).

Independent pharmacies are associated with perceptions of lower-quality health information (*p* = 0.077), while experienced pharmacists are less likely to provide advice on self-medication risks (*p* = 0.014). Additionally, chain pharmacies are more likely to emphasize the importance of public health education programs (*p* = 0.026). Lastly, the COVID-19 pandemic has significantly influenced self-medication patterns, with experienced pharmacists noting substantial changes in these practices (*p* = 0.018).

## 5. Discussion

Self-medication can provide benefits, such as reducing the burden on healthcare systems by allowing patients to manage minor health issues independently. However, it carries significant risks, including incorrect diagnoses, improper medication use, and potentially dangerous drug interaction [34,38]. These risks are heightened by uncontrolled access to unverified online medical information, which can lead to misguided self-treatment decisions and improper use of over-the-counter medications [13,39].

Pharmacists, being frontline health professionals, play a pivotal role in guiding self-medication practices. Their responsibilities include assessing the appropriateness of medications and providing clear guidance to minimize risks. This role could be further enhanced by developing targeted education programs tailored to different demographic needs, focusing on safe self-medication behaviors and promoting informed health choices [8,40]. For example, pharmacists could provide easy-to-understand brochures, host educational workshops, or use social media campaigns to distribute accurate information.

While self-medication can temporarily ease pressure on healthcare services by enabling quick access to treatment for minor conditions, it is not a sustainable practice without appropriate regulation and comprehensive health education. Effective interventions may include public awareness campaigns, health literacy programs in schools, and digital tools that provide reliable information on self-care and proper medication use. Active collaboration among healthcare providers—including doctors, nurses, and pharmacists—and public health officials is crucial to promote responsible self-medication and reduce associated risks [41,42,43,44].

### 5.1. Synthesis of Results and Comparison with the Specialized Literature

The results of our study, which investigated self-medication behaviors among urban and rural populations, focusing on the perceptions and practices of pharmacists in managing this phenomenon, partially or fully confirmed the hypotheses initially formulated. First, Hypothesis 1 (H1), which proposed a correlation between the frequency of self-medication and the perceived severity of the condition, was confirmed. Minor ailments, such as colds and mild pains, led to an increased demand for self-medication, with 92.3% of pharmacists reporting daily requests for such conditions, analgesics being the most requested (23.9%). On the other hand, while self-medication for more severe conditions was less frequent (59.3%), the frequent use of antibiotics without a prescription (19.1%) raises significant concerns about their overuse and the impact on public health.

Hypothesis 2 (H2), which suggested a higher predisposition to self-medication in urban areas compared to rural areas, was also confirmed. In total, 85% of the interviewed pharmacists worked in urban areas, where access to medical information and healthcare services is easier. This greater accessibility may explain the higher prevalence of self-medication in cities, although the differences with rural areas were not as pronounced as initially anticipated.

Regarding Hypothesis 3 (H3), our study demonstrated that pharmacists with more extensive professional experience are more reluctant to recommend self-medication, being more aware of the associated risks. Pharmacists with over 25 years of experience more frequently refused unjustified requests for medication (34%) compared to their younger colleagues, who were more willing to respond to such requests.

Although Hypothesis 4 (H4), which emphasized the role of public education in reducing the prevalence of self-medication, was only partially confirmed, the results indicate that the general level of awareness of the risks remains low. Approximately 30% of pharmacists reported a low awareness of risks among patients, highlighting the need for more effective and widespread educational campaigns to address self-medication behaviors.

Therefore, our study confirms most of the research hypotheses and highlights the need for stricter educational and regulatory measures to reduce self-medication, especially in the case of severe conditions. Additionally, pharmacists play a crucial role in educating patients and managing this phenomenon, contributing to the responsible use of over-the-counter medications.

When comparing these results with the existing literature, our study aligns with much of the previous research while also providing distinct perspectives. Regarding the correlation between the frequency of self-medication and the perceived severity of the condition (H1), numerous studies confirm that patients are more likely to self-medicate for minor conditions, frequently using analgesics and anti-inflammatories [45,46,47]. However, the use of antibiotics without a prescription for more severe conditions remains a major concern, confirmed by other research [48,49,50].

The hypothesis regarding the differences between urban and rural environments (H2) is also supported by other studies, which show that self-medication is more common in urban areas due to easier access to medications and medical information [51,52,53]. The influence of drug advertising is another factor contributing to this difference [54,55].

Regarding the influence of pharmacists’ experience on the recommendation of self-medication (H3), the specialized literature supports our findings. Pharmacists with more professional experience tend to refuse requests for self-medication more frequently and instead encourage patients to seek medical consultations. This behavior highlights their deeper understanding of the potential risks associated with self-medication, such as misdiagnoses, inappropriate drug use, or adverse interactions [56,57].

Public education on the risks of self-medication (H4) remains an area that requires greater attention. Although education contributes to reducing the prevalence of self-medication, the overall level of awareness remains low, as confirmed by other studies [58,59]. Studies suggest that educating the public about the risks of self-medication can significantly reduce the incidence of this behavior, especially in cases of antibiotic misuse and other medications without prescriptions [46,50,60].

Thus, the results of our study are in line with the specialized literature, but emphasize the importance of additional measures in educating the public and regulating self-medication.

### 5.2. Interpretation of Results and Implications for Medical Practice and Public Health

The results of our study provide a detailed understanding of the self-medication phenomenon within the population, highlighting general trends and emphasizing the pivotal role pharmacists play in managing this behavior. Below, we interpret the findings and explore their implications for medical practice and public education.

Self-medication for minor ailments emerged as a frequent behavior, as evidenced by the data presented in Table 2. The daily demand for analgesics and cold medications reflects a strong inclination among patients to treat minor symptoms independently, without consulting a healthcare professional [61]. Although this practice is generally viewed as harmless in the case of mild conditions, there is a risk that inappropriate use of medications may lead to complications or mask underlying chronic conditions that remain undiagnosed [62]. This raises significant concerns regarding the potential long-term effects of such self-medication practices [63].

To mitigate the associated risks, pharmacists must actively engage in educating patients on the proper use of over-the-counter medications [64]. A firm stance from pharmacists is essential, not only in guiding patients towards the correct dosages and warning them of possible side effects but also in encouraging medical consultation when symptoms persist or worsen. On a broader level, national awareness campaigns could play a crucial role in informing the public about the responsible use of medication, while also promoting regular health check-ups to ensure the early detection and treatment of more serious conditions [65].

A significant issue that emerged from the study, as shown in Table 2, is the frequent use of antibiotics without a medical prescription for more severe conditions [66]. This is a global concern, as the overuse and misuse of antibiotics contribute to the rise in antibiotic resistance, which poses a grave threat to public health [67]. Unregulated antibiotic consumption not only diminishes their effectiveness but also exposes patients to serious health risks, including the development of resistant bacterial strains and the potential for severe complications [68].

Pharmacists are key players in addressing this issue [69]. It is imperative that they adhere strictly to legal regulations by refusing to dispense antibiotics without a valid prescription, while simultaneously educating patients about the dangers of antibiotic misuse. Strengthened regulatory frameworks are needed to restrict access to antibiotics, ensuring that they are only available by prescription [70]. In conjunction with this, community-level educational programs could raise awareness about the risks of inappropriate antibiotic use and promote safe and effective treatment practices.

Our study also confirmed that urban populations are more inclined toward self-medication than rural populations, as indicated by the data in Table 1, where the majority of pharmacists reported working in urban settings. This can be attributed to easier access to medical information and medications in urban areas, which encourages more frequent self-medication behavior [71]. In contrast, the limited availability of healthcare services in rural areas may lead to self-medication for more severe conditions, though the overall frequency of self-medication remains lower compared to urban areas [72].

Addressing this disparity requires tailored interventions based on the specific needs of urban and rural populations [73]. In urban areas, educational efforts should focus on raising awareness about the risks of self-medicating for more serious conditions, particularly the misuse of antibiotics [74]. In rural areas, improving access to accurate medical information and medications is essential. This could be achieved through mobile education and prevention campaigns, as well as facilitating access to regular medical consultations for isolated communities [75].

The education and professional experience of pharmacists were shown to significantly influence how they manage self-medication requests. More experienced pharmacists demonstrated a greater reluctance to recommend self-medication, as seen in Table 5. However, only a fraction of pharmacists (37.3%) reported the existence of clear protocols for evaluating over-the-counter medication requests, as indicated in Table 6. This highlights an inconsistent application of standardized procedures across pharmacies.

To address this, it is crucial to implement clear, nationwide protocols for pharmacists in assessing self-medication requests [76]. These protocols should be supported by continuous training programs aimed at strengthening pharmacists’ understanding of the risks associated with self-medication and providing them with effective communication strategies to refuse inappropriate requests [77]. Additionally, it is important to ensure that the information provided to patients in pharmacies is of a consistent and high standard, offering accurate and reliable guidance [78].

The COVID-19 pandemic had a profound impact on self-medication behavior, as reflected in Table 4. A significant proportion of pharmacists (67%) reported an increase in self-medication during the pandemic, attributed to both restricted access to healthcare services and patients’ fear of visiting medical facilities [79,80]. This behavior increases the likelihood of complications, particularly if patients self-administer medications that are inappropriate for their conditions or lack proper understanding of their ailments [81].

To prevent excessive self-medication during crises, it is vital for health authorities and professionals to develop and implement clear communication strategies that encourage patients to seek safe and appropriate medical care [79,80,82]. Pharmacies should be better equipped to manage increased demands for self-medication during such periods, ensuring that patients have access to reliable information regarding their treatment options [83].

In summary, the findings of our study underline the complexity of self-medication behaviors and the critical role of pharmacists in mitigating the risks associated with this practice. Pharmacists must be proactive in educating the public and ensuring that self-medication is practiced responsibly. At the same time, regulatory measures should be strengthened to safeguard against the misuse of medications, particularly antibiotics. Public education campaigns, tailored to the needs of both urban and rural populations, will be instrumental in promoting safer healthcare practices and improving overall public health outcomes. Considering that pharmacists in Romania currently have limited access to patients’ clinical histories, a health policy allowing them regulated access to relevant medical information could help reduce self-medication by enabling more informed and personalized recommendations for patients. Furthermore, in Romania, there are currently no regulations that allow pharmacists to interact directly with doctors in the case of a patient engaging in self-medication. The role of pharmacists is limited to recommending that the patient consult a doctor when they believe the patient’s condition requires specialist intervention. This lack of regulation highlights the need for policies that facilitate communication between pharmacists and doctors to ensure safer and more effective patient care.

### 5.3. Study Limitations and Implications for Future Research

While this study has offered valuable insights into self-medication behaviors and the critical role pharmacists play in managing them, several limitations need to be acknowledged to ensure a comprehensive understanding of the results and the broader context in which they were obtained. One of the main factors that may limit the generalization of these findings is the relatively small sample size and the focus on urban pharmacists. The majority of respondents (85%, according to Table 1) are from urban areas, which may lead to an underrepresentation of practices and perceptions in rural settings. Although the study included pharmacists from both environments, the uneven distribution might influence conclusions related to self-medication behaviors in rural areas. Therefore, future research would benefit from expanding the sample to include a larger proportion of rural pharmacists, allowing for a clearer understanding of the phenomenon across various geographical contexts. Another limitation of the study lies in the fact that the professional experience of pharmacists was evaluated exclusively based on their years of activity, without considering other relevant factors such as participation in continuing education programs, involvement in specific clinical activities, or other forms of professional training, which could provide a more nuanced perspective on this aspect.

Future research directions should also explore the long-term effects of self-medication, particularly regarding the excessive use of antibiotics or other over-the-counter medications without medical supervision. A deeper analysis of the health consequences, such as the rise in antibiotic resistance or complications caused by improper medication use, is essential. This could involve longitudinal studies tracking the health outcomes of patients who frequently engage in self-medication practices.

Moreover, regulatory policies play a significant role in controlling access to medications, and further research could examine the impact of changes in legislation related to over-the-counter drugs on self-medication behaviors. For example, studies analyzing the effects of stricter policies on the dispensing of antibiotics would provide valuable insights into the effectiveness of these measures in curbing medication misuse. Investigating how pharmacies enforce these regulations and how these practices differ across independent and chain pharmacies (as noted in Table 6) would also contribute to a better understanding of the broader regulatory landscape.

Education is another crucial factor in reducing inappropriate self-medication, and future studies should assess the effectiveness of various public education campaigns designed to promote the responsible use of medications. Experimental studies could evaluate the impact of different educational interventions, such as digital education, media campaigns, or school-based programs, on improving public awareness and modifying self-medication behaviors.

Finally, the ongoing impact of the COVID-19 pandemic on self-medication behaviors remains an important area of research. As Table 4 indicates, the pandemic significantly altered these behaviors, primarily due to restricted access to healthcare services. Future research should investigate whether these behaviors have persisted as restrictions have eased and life has returned to a more stable post-pandemic state, or if self-medication trends have reverted to pre-pandemic levels.

## 6. Conclusions

The present study provided a detailed perspective on the phenomenon of self-medication, focusing on the role of pharmacists and the demographic influences on this behavior. The results confirmed the hypothesis that self-medication is more frequent in the case of minor ailments and highlighted the significant risk of antibiotic misuse without a medical prescription. It was also observed that experienced pharmacists are more likely to responsibly manage unjustified medication requests, though there is still room for improvement in procedures and public education.

This study contributes to a better understanding of self-medication and the essential role pharmacists play in educating patients and preventing the improper use of medications. It also highlights the need for additional regulatory and educational measures to reduce uncontrolled self-medication, particularly regarding antibiotics.

By identifying these trends and challenges, our research provides a solid foundation for the development of future intervention and education strategies aimed at improving public health and promoting the responsible use of over-the-counter medications.

## Figures and Tables

**Table 1:** Demographic characteristics of pharmacists.

Category	Indicators: Distribution (%)
Professional Experience	<1 year: 2.6% 1–5 years: 18.6% 6–10 years: 14.0% 11–15 years: 21.6% 16–20 years: 15.3% 21–25 years: 14.3% >25 years: 13.3%
Working Environment	Urban: 85.0% Rural: 15.0%
Age	<30 years: 16.0% 30–35 years: 16.6% 36–40 years: 18.3% 41–45 years: 19.6% 46–50 years: 14.0% >51 years: 15.3%
Education Level	Pre-university: 1.6% * University: 61.6% Postgraduate: 36.6%
Type of Pharmacy	Independent: 42.0% Pharmacy chain: 58.0%

* Pharmacy assistants.

**Table 2:** Dynamics of self-medication requests, types of medicines requested, and common associated conditions.

Type of Request/Medical Condition	Frequency of Request/Condition (%)	Most Common Medicines (%)	Chi-Square	*p*-Value
Minor Conditions (self-medication)	Daily: 92.3%	Analgesics: 23.9%	130.680	<0.001
Serious Conditions (self-medication)	Daily: 59.3%	Antibiotics: 19.1%	32.013	<0.001
Cold and Flu	Very Frequently (65.0%)	Anti-inflammatories: 23.2%	110.413	<0.001
Gastrointestinal Conditions	Frequently (36.3%)	Antipyretics: 11.8%	9.720	0.002
Sleep or Stress Disorders	Moderately (34.0%)	Anxiolytics: 7.2%	75.000	<0.001
COVID-19	Occasionally (25.6%)	Dietary supplements: 14.1%	0.120	0.729

**Table 3:** Perceptions of access to medication information, influences on self-medication, and quality of pharmacy information.

Category	Indicator	Distribution (%)	Chi-Square	*p*-Value
Access to medication information	Yes	26.3%	1.58	>0.05
	No	30.3%		
	Sometimes	43.3%		
			**Mean (std. dev.)**	
Influences on self-medication phenomenon	Medication advertisements (Very much)	78.0%	4.70 (0.63)	
	Information from social networks (Very much)	50.0%	4.20 (0.97)	
Quality of information available in pharmacies	Very high	38.0%	4.17 (0.80)	
	High	45.0%		
	Medium	14.0%		
	Low	2.3%		
	Very low	0.6%		

**Table 4:** Perceptions of the Impact of the COVID-19 Pandemic on Self-Medication.

Indicator	Distribution (%)	Chi-Square	*p*-Value
Impact of the pandemic on self-medication	Yes: 67.0%	0.261	>0.05
	No: 14.3%		
	Not sure: 18.6%		

**Table 5:** Understanding of non-prescription medication use, risk awareness, encouragement of medical consultation, and pharmacists’ comfort in managing self-medication.

Category	Indicator	Distribution (%)	Chi-Square	*p*-Value
Understanding the necessity of non-prescription medications	<25%	36.3%	1.05	>0.05
	25–50%	43.3%		
	51–75%	18.0%		
	>75%	2.3%		
			**Mean (std. dev.)**	
Awareness of the risks of self-medication	Very low awareness	29.0%	2.36 (1.20)	
	Low awareness	30.3%		
	Moderate awareness	24.3%		
	High awareness	8.3%		
	Very high awareness	8.0%		
Encouragement of medical consultation for serious conditions	Very high	89.0%	4.85 (0.46)	
	High	8.0%		
	Moderate	2.3%		
	Low	0.6%		
	Never	0.0%		
Pharmacists’ comfort in selling inappropriate medications	Very comfortable	52.6%	4.12 (1.15)	
	Comfortable	22.0%		
	Moderately comfortable	16.3%		
	Uncomfortable	3.0%		
	Very uncomfortable	6.0%		

**Table 6:** Existence of procedures for evaluating the necessity of non-prescription medications and managing requests for inappropriate medications for self-medication.

Category	Indicator	Distribution (%)	Chi-Square	*p*-Value
Existence of procedures for evaluating the necessity of medications	Yes	37.3%	0.92	>0.05
	No	14.0%		
	Only in some cases	48.6%		
Managing requests for inappropriate medications	Polite refusal	5.0%	0.58	>0.05
	Medical consultation recommendation	56.6%		
	Risk education	37.6%		
	Other methods	0.6%		

**Table 7:** Pharmacists’ approaches to managing risky self-medication and proposed strategies for improving the management of self-medication.

Category	Indicator	Distribution (%)
Pharmacists’ approaches to managing risky self-medication	Polite refusal and redirection to a doctor	34.0%
	Explanation of risks	29.0%
	Request for a medical prescription	18.0%
	Evaluation and personalized advice	19.0%
Proposed strategies for improving self-medication management	Education and information about the risks of self-medication	40.0%
	Regulation and control of sales, including prescription requirements	30.0%
	More active involvement of healthcare professionals (pharmacists, doctors)	20.0%
	Restriction of medication advertisements	10.0%

**Table 8:** Key demographic and professional factors influencing self-medication practices.

Dependent Variable	Independent Variable	Unstandard. Coeffic. (B)	Standardized Coefficient (Beta)	*p*-Value
Self-medication for sleep/stress disorders	Type of pharmacy (independent vs. chain)	0.302	0.157	0.006
Self-medication for COVID-19	Pharmacy location (rural vs. urban)	−0.470	−0.130	0.028
	Education level (university vs. postgraduate)	−0.294	−0.116	0.044
Self-medication for colds and flu	Age range	−0.089	−0.188	0.001
Impact of medication advertisements on self-medication	Professional experience	−0.074	−0.200	0.000
	Education level (university vs. postgraduate)	−0.149	−0.119	0.036
Role of social networks in self-medication decisions	Age range	−0.060	−0.101	0.080
Frequency of citing online information for medication requests	Professional experience	−0.080	−0.161	0.005
Quality of health information available in pharmacy	Type of pharmacy (independent vs. chain)	−0.166	−0.102	0.077
Providing advice or materials on self-medication risks	Professional experience	−0.075	−0.141	0.014
Necessity of public health education programs	Type of pharmacy (independent vs. chain)	0.092	0.128	0.026
Impact of the COVID-19 pandemic on self-medication	Professional experience	0.062	0.136	0.018

## Data Availability

Data can be requested from the corresponding author.
